# High critical current density and high-tolerance superconductivity in high-entropy alloy thin films

**DOI:** 10.1038/s41467-022-30912-5

**Published:** 2022-06-11

**Authors:** Soon-Gil Jung, Yoonseok Han, Jin Hee Kim, Rahmatul Hidayati, Jong-Soo Rhyee, Jung Min Lee, Won Nam Kang, Woo Seok Choi, Hye-Ran Jeon, Jaekwon Suk, Tuson Park

**Affiliations:** 1 Center for Quantum Materials and Superconductivity (CQMS), Sungkyunkwan University, Suwon, 16419 Republic of Korea; 2 Department of Physics, Sungkyunkwan University, Suwon, 16419 Republic of Korea; 3 Department of Applied Physics, Integrated Education Institute for Frontier Science and Technology (BK 21 Four), Kyung Hee University, Yongin, 17104 Republic of Korea; 4 Institute of Natural Science, Kyung Hee University, Yongin, 17104 Republic of Korea; 5 Korea Multi-purpose Accelerator Complex, Korea Atomic Energy Research Institute, Gyeongju, Gyeongbuk 38180 Republic of Korea

**Keywords:** Superconducting properties and materials, Surfaces, interfaces and thin films

## Abstract

High-entropy alloy (HEA) superconductors—a new class of functional materials—can be utilized stably under extreme conditions, such as in space environments, owing to their high mechanical hardness and excellent irradiation tolerance. However, the feasibility of practical applications of HEA superconductors has not yet been demonstrated because the critical current density (*J*_c_) for HEA superconductors has not yet been adequately characterized. Here, we report the fabrication of high-quality superconducting (SC) thin films of Ta–Nb–Hf–Zr–Ti HEAs via a pulsed laser deposition. The thin films exhibit a large *J*_c_ of >1 MA cm^−2^ at 4.2 K and are therefore favorable for SC devices as well as large-scale applications. In addition, they show extremely robust superconductivity to irradiation-induced disorder controlled by the dose of Kr-ion irradiation. The superconductivity of the HEA films is more than 1000 times more resistant to displacement damage than that of other promising superconductors with technological applications, such as MgB_2_, Nb_3_Sn, Fe-based superconductors, and high-*T*_c_ cuprate superconductors. These results demonstrate that HEA superconductors have considerable potential for use under extreme conditions, such as in aerospace applications, nuclear fusion reactors, and high-field SC magnets.

## Abstract (web summary)

Thin-film high-entropy alloy (HEA) superconductors have recently attracted a lot of attention, but their critical current density and potential usefulness in engineering applications has remained unclear. Here, the authors fabricate HEA films with remarkably high critical current density and resistance to radiation damage.

## Introduction

High-entropy alloys (HEAs), which are typically composed of multiple metallic elements, open new avenues for the design of novel functional materials because they have superior physical and mechanical properties to conventional alloys^1–8^. In general, HEAs have simple body-centered cubic (bcc), face-centered cubic (fcc), and hexagonal close-packed (hcp) structures^3,4^, although five or more elements randomly occupy one available crystallographic position, with each element having an atomic fraction between 5 and 35%^1,3^. Thermodynamically, a single phase solid solution in HEAs is considered due to a dominance of the entropy of mixing (Δ*S*_mix_) in the Gibbs free energy difference, Δ*G*_mix_ = Δ*H*_mix_ − *T*Δ*S*_mix_, where *T* is the temperature and Δ*H*_mix_ is the enthalpy of mixing that contributes to phase separation or the formation of multi-component alloys^3,4^. Because the elements have slightly different atomic sizes, the novel physical properties and high mechanical hardness of HEAs are believed to result from their large atomic disorder^3,4,8^.

The superconductivity of HEAs was discovered in 2014 in Ta–Nb–Hf–Zr–Ti multi-principal element alloy, attracting considerable interest in their pairing mechanism; generally, a high level of disorder in crystalline superconductors limits the formation of Cooper pairs partially owing to the decrease in the density of states or the increase in effective Coulomb repulsion between paired electrons^9,10^. In addition, the unique properties of HEAs, such as their high hardness, high strength, and excellent irradiation tolerance, are advantageous for use under extreme conditions such as those in aerospace applications, irradiation environments, and superconducting (SC) rotating machines^2–4,6,11^. Recently, the robust superconductivity of Ta–Nb–Hf–Zr–Ti HEAs under extremely high pressures of approximately 190 GPa has been reported^5^, where the unit cell volume of Ta–Nb–Hf–Zr–Ti HEAs was expected to be considerably compressed by ~53%. Since the lattice constant of a crystal is closely related to its electronic structure, we believe that HEA superconductors are not only interesting for fundamental studies of pairing mechanism, but also promising for applications under extreme conditions^12^. However, the critical current density (*J*_c_), i.e., the maximum current density that can conduct electric current without any power dissipation, was reported to be 0.01–10 kA cm^−2^ for Co–Ni–Cu–Rh–Ir–Zr and Ta–Nb–Hf–Zr–Ti HEA superconductors. This value is extremely low for practical applications^13,14^.

Here, we report high-*J*_c_ Ta–Nb–Hf–Zr–Ti HEA SC thin films fabricated by pulsed laser deposition (PLD) and the extraordinarily robust superconductivity against irradiation-induced disorder verified using 200 keV Kr-ion irradiation. The HEA SC thin films were deposited on *c*-cut Al_2_O_3_ substrates over a wide range of substrate temperatures (*T*_s_) from 270 to 620 °C. A film deposited at *T*_s_ = 520 °C showed the highest SC transition temperature (*T*_c_ = 7.28 K) and the largest *J*_c_ (>1 MA cm^−2^) at 4.2 K, which is promising for SC devices as well as large-scale applications. Moreover, the superconductivity of Ta–Nb–Hf–Zr–Ti HEA SC thin films was found to be approximately 1000 times more resistant to irradiation damage than that of other promising SC materials, including high-*T*_c_ cuprate superconductors. Taken together, these discoveries suggest that the HEA SC thin films possess considerable potential for technological applications under extreme conditions.

## Results and discussion

### Crystal structure and SC transition in HEA thin films

**Fig. 1:**
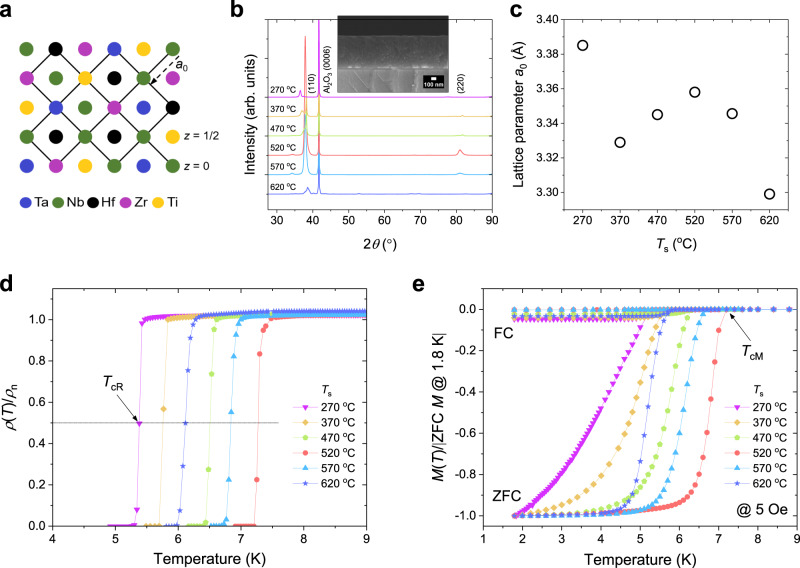
Crystal structure and superconducting (SC) transition temperature of Ta–Nb–Hf–Zr–Ti HEA SC thin films. **a** Schematic of the bcc lattice with randomly distributed atoms of the Ta_1/6_Nb_2/6_Hf_1/6_Zr_1/6_Ti_1/6_ HEA superconductor. **b** XRD results for Ta–Nb–Hf–Zr–Ti HEA SC thin films deposited on a *c*-cut Al_2_O_3_ substrate at *T*_s_ = 270, 370, 470, 520, 570, and 620 °C, indicating a (110) preferred orientation. The inset shows a cross-sectional SEM image for the film fabricated at *T*_s_ = 520 °C. **c** The lattice parameter (*a*_0_) of HEA SC thin films with respect to substrate temperature *T*_s_. **d**, **e** Temperature dependence of the electrical resistivity (*ρ*) and magnetization (*M*) for the Ta–Nb–Hf–Zr–Ti HEA SC thin films, respectively. Here, *ρ*(*T*) and *M*(*T*) were normalized to the *ρ* at the *T*_c_ onset (*ρ*_n_) and the absolute zero-field-cooled (ZFC) *M* value at 1.8 K, respectively, for comparison. The field-cooled (FC) and ZFC *M*(*T*) were measured at 5 Oe (applied perpendicularly to the film plane).

Figure 1a shows a schematic of the bcc crystal structure of Ta–Nb–Hf–Zr–Ti superconductors with a compositional ratio of Ta:Nb:Hf:Zr:Ti = 1:2:1:1:1, where each color represents the expected atomic fraction of each element occupying one crystallographic site. For instance, the Nb atom, which is indicated by the green color, accounts for a fraction of 1/3, whereas Ta, Hf, Zr, and Ti each account for 1/6. The X-ray diffraction (XRD) patterns of *θ*–2*θ* scans of the Ta–Nb–Hf–Zr–Ti HEA SC thin films fabricated on *c*-cut Al_2_O_3_ substrates indicated that all the films with the bcc crystal structure had a preferred orientation of (110), regardless of the substrate temperature (*T*_s_), as shown in Fig. 1b. The inset of Fig. 1b shows a cross-sectional scanning electron microscopy (SEM) image of the film fabricated at *T*_s_ = 520 °C with a thickness of approximately 700 nm. The lattice parameter *a*_0_ with respect to *T*_s_ for the HEA SC thin films are summarized in Fig. 1c, and the film deposited at *T*_s_ = 520 °C, which exhibits the highest XRD peak intensity, has *a*_0_ = 3.358 Å, which is similar to that of bulk Ta_1/6_Nb_2/6_Hf_1/6_Zr_1/6_Ti_1/6_ HEA superconductors^13^. The slight peak shift and low intensity for the thin films fabricated at *T*_s_ values other than 520 °C are more closely related to the substrate temperature than the compositional ratio of the films^15,16^ (see Supplementary Table 1 and Supplementary Figs. 1–3).

Figure 1d presents the temperature dependence of the electrical resistivity (*ρ*) of the Ta–Nb–Hf–Zr–Ti HEA SC thin films grown at *T*_s_ = 270, 370, 470, 520, 570, and 620 °C, where *ρ*(*T*) is normalized to the *ρ* value at the *T*_c_ onset (*ρ*_n_) for comparison. All the films exhibited a sharp SC transition, and the film fabricated at 520 °C showed the highest SC transition temperature (*T*_c_ = 7.28 K). Here, the *T*_c_ from the *ρ*(*T*) curves (*T*_cR_) was determined by the 50% transition of *ρ*_n_, as indicated by the solid line and arrow in Fig. 1d. The bulk SC transition for the HEA SC thin films, which was investigated using zero-field-cooled (ZFC) and field-cooled (FC) dc magnetization (*M*), also showed similar trends to the *ρ*(*T*) curves, as shown in Fig. 1e. All the films exhibited clear Meissner signals and sharp SC transitions in the ZFC *M*(*T*) curves, reflecting their high quality. Here, *M*(*T*) was normalized to the absolute ZFC *M* value at 1.8 K for comparison, and the *T*_c_ from the *M*(*T*) curves (*T*_cM_) was determined by the irreversible points of the ZFC and FC *M*(*T*) curves, as indicated by the arrow representing the film deposited at the optimal *T*_s_ = 520 °C.

**Fig. 2:**
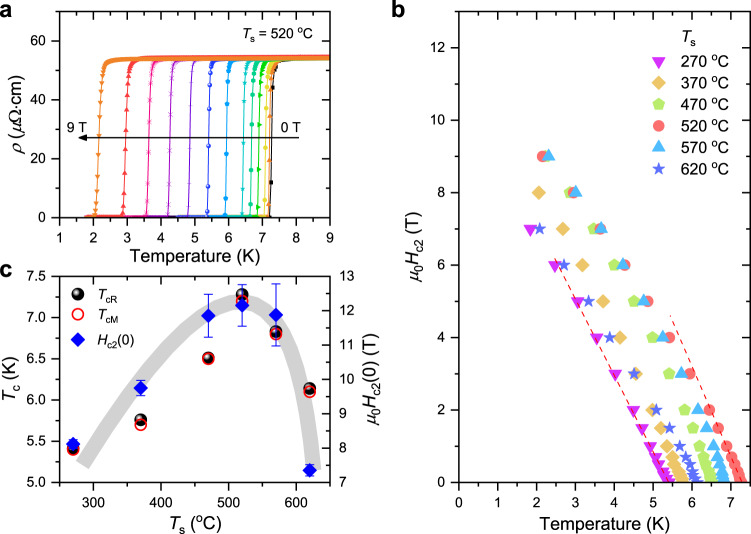
Upper critical fields and SC transition temperatures of HEA SC thin films with respect to the growth temperature. **a** Representative *ρ*(*T*) curves under various magnetic fields ranging from 0 to 9 T for the HEA SC thin film fabricated at the optimal *T*_s_ = 520 °C, where the direction of the applied magnetic field was perpendicular to the *ab* plane of the films. **b** Upper critical field (*μ*_0_*H*_c2_) as a function of the temperature for the HEA SC thin films, determined by 50% of the SC transition from the *ρ*_n_ at each magnetic field. Red dashed lines for *T*_s_ = 270 and 520 °C indicate representative linear fits for the estimation of d*H*_c2_/d*T* near *T*_c_. **c** *T*_c_ and *μ*_0_*H*_c2_ at zero Kelvin, *μ*_0_*H*_c2_(0), with respect to the substrate temperature *T*_s_. Here, *T*_cR_ and *T*_cM_ were determined by the 50% transition of *ρ*_n_ and the irreversible points of the ZFC and FC *M*(*T*) curves, respectively, and the *μ*_0_*H*_c2_(0) was estimated using the Werthamer–Helfand–Hohenberg model. *T*_c_ and *μ*_0_*H*_c2_(0) exhibit a similar behavior with respect to *T*_s_, implying that the high *T*_c_ of HEA SC thin films leads to a large *μ*_0_*H*_c2_(0). The error bars on the *μ*_0_*H*_c2_(0) reflect the uncertainties in the linear fitting of d*H*_c2_/d*T* around *T*_c_. Half-transparent shades are guides to the eyes.

The dependence of the magnetic field of the SC phase transition is selectively displayed for the Ta–Nb–Hf–Zr–Ti HEA SC thin film deposited at the optimal *T*_s_ = 520 °C in Fig. 2a, where various magnetic fields from 0 to 9 T were applied perpendicularly to the film plane (see also Supplementary Fig. 4). Although the magnitude of the applied magnetic field increased, the SC transition of the HEA SC thin films did not broaden considerably, revealing that the HEA superconductor has a strong vortex pinning strength^17,18^. In general, because the number of vortices is proportional to the magnetic field and the vortex motion generates an electric field, an increase in the magnetic field results in a broad SC transition in type-II superconductors with weak vortex pinning strengths^19,20^.

Figure 2b shows the upper critical field (*μ*_0_*H*_c2_) as a function of the temperature for the HEA SC thin films, where *μ*_0_*H*_c2_(*T*) was obtained from the *ρ*(*T*, *H*) curves using the same criterion that was employed for *T*_cR_ (50% of *ρ*_n_). The red dashed lines indicate the linear slope of *H*_c2_ near *T*_c_ for the films deposited at *T*_s_ = 270 and 520 °C, where (d*H*_c2_/d*T*)_*T*=*T*c_ is −2.17, −2.44, −2.63, −2.41, −2.51, and −1.73 T K^−1^ for the HEA SC thin films deposited at *T*_s_ = 270, 370, 470, 520, 570, and 620 °C, respectively. The upper critical field at zero Kelvin, *μ*_0_*H*_c2_(0), was evaluated using the Werthamer–Helfand–Hohenberg (WHH) formula in the dirty limit:^21^

1 $${\mu }_{0}{{H}_{{{{{{\rm{c}}}}}}2}}^{{{{{{\rm{WHH}}}}}}}(0)=-0.693{T}_{{{{{{\rm{c}}}}}}}{({{{{{\rm{d}}}}}}{H}_{{{{{{\rm{c}}}}}}2}/{{{{{\rm{d}}}}}}T)}_{T{{{{{\rm{c}}}}}}}.$$

The estimated *μ*_0_*H*_c2_(0) values of the HEA SC thin films and the SC transition temperatures determined from the electrical resistivity (*T*_cR_) and magnetization (*T*_cM_) are displayed in Fig. 2c. It is observed that *μ*_0_*H*_c2_(0) with respect to *T*_s_ exhibits a similar tendency to *T*_c_, indicating that a higher *T*_c_ can induce a larger *μ*_0_*H*_c2_(0) of the HEA SC thin films. However, the *μ*_0_*H*_c2_(0) of the thin films fabricated at temperatures above the optimal *T*_s_ = 520 °C showed a more rapid reduction compared to the decrease in *T*_c_. Despite the high *T*_c_, small slope values of (d*H*_c2_/d*T*)_*T*=*T*c_ have also been reported in (TaNb)_1−*x*_(HfZrTi)_*x*_ HEA superconductors by controlling the mixing entropy or application of pressure^5,15,16^. Typically, because the SC coherence length (*ξ*) in dirty type-II superconductors is proportional to the mean free path (*l*) of the charge carriers, *H*_c2_(0) can be improved by adjusting the disorder level^22,23^.

### Critical current density of HEA SC thin films

**Fig. 3:**
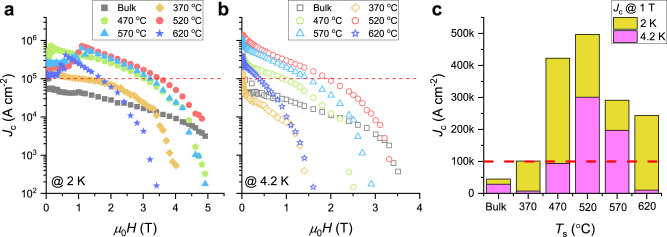
Magnetic field dependence of the critical current density for Ta–Nb–Hf–Zr–Ti HEA SC thin films. **a**, **b** Magnetic field dependence of the critical current density (*J*_c_) for HEA SC thin films at 2.0 and 4.2 K, respectively. All the films show remarkably large *J*_c_ values at 0 T compared with that of the bulk HEA SC sample, as well as promising field performance. The low-field *J*_c_ at 2.0 K is lower than that at 4.2 K because of the considerable flux jump at 2.0 K. The red dashed line marks *J*_c_ = 100 kA cm^−2^, which is a common benchmark for large-scale applications such as high-field SC magnets. **c** Relative levels of *J*_c_ at 2.0 and 4.2 K at 1 T show that HEA superconductors are available for widespread applications.

Figure 3a and b show the magnetic field dependence of the critical current density (*J*_c_) at 2.0 K and 4.2 K, respectively, for Ta–Nb–Hf–Zr–Ti HEA SC bulk and SC thin films deposited at different temperatures. The *J*_c_ data for the bulk sample are for the high-quality Ta_1/6_Nb_2/6_Hf_1/6_Zr_1/6_Ti_1/6_ superconductor with *T*_cM_ = 7.8 K used as the target for the deposition of HEA SC thin films in this study (see Supplementary Fig. 5a). All the films exhibited substantially larger *J*_c_ values than the bulk sample—particularly those grown at *T*_s_ = 470, 520, and 570 °C (see also Supplementary Fig. 5). For example, the magnitudes of *J*_c_ for a film deposited at the optimal *T*_s_ = 520 °C, which had *J*_c_ > 1 MA cm^−2^, were approximately 820 and 790% larger than those of the bulk sample at 2.0 K (@ 3.4 T) and 4.2 K (@ 2 T), respectively. Here, the magnitude of *J*_c_(@ 0 T) of the HEA SC thin films is comparable to that of the most widely used commercial superconductor NbTi alloy^24–26^. A higher *J*_c_(@ 0 T), i.e., self-field *J*_c_, in SC thin films compared to bulk samples is commonly observed for most SC materials, including MgB_2_ and high-*T*_c_ cuprates^27,28^. This is thought to be because the self-field *J*_c_ is confined to the surface area associated with lower critical field (*H*_c1_) and London penetration depth (*λ*) rather than distributed over the entire cross-sectional area^28,29^. The strong field performance of *J*_c_ of the HEA SC thin films is considered to be closely associated with the intrinsic internal disorder caused by the slightly different atomic sizes of the constituent atoms (see Supplementary Fig. 6). The large reduction in the low-field *J*_c_ at 2.0 K resulted from the large flux jump due to thermal instability (see Supplementary Fig. 7)^30,31^, indicating that the *J*_c_ of the HEA SC thin films can be further improved^32^. The red dashed lines indicate *J*_c_ = 0.1 MA cm^−2^, which is a common benchmark for large-scale practical applications of superconductors such as high-field SC magnets^33^. Relative magnitudes of *J*_c_ at 2.0 and 4.2 K at 1 T were described in Fig. 3c. The large *J*_c_ values over a wide range of film growth temperatures suggest that HEA superconducting thin films not only have considerable potential for SC devices, but also can replace conventional SC alloys in practical engineering applications.

### Effect of ion irradiation on HEA SC thin films

**Fig. 4:**
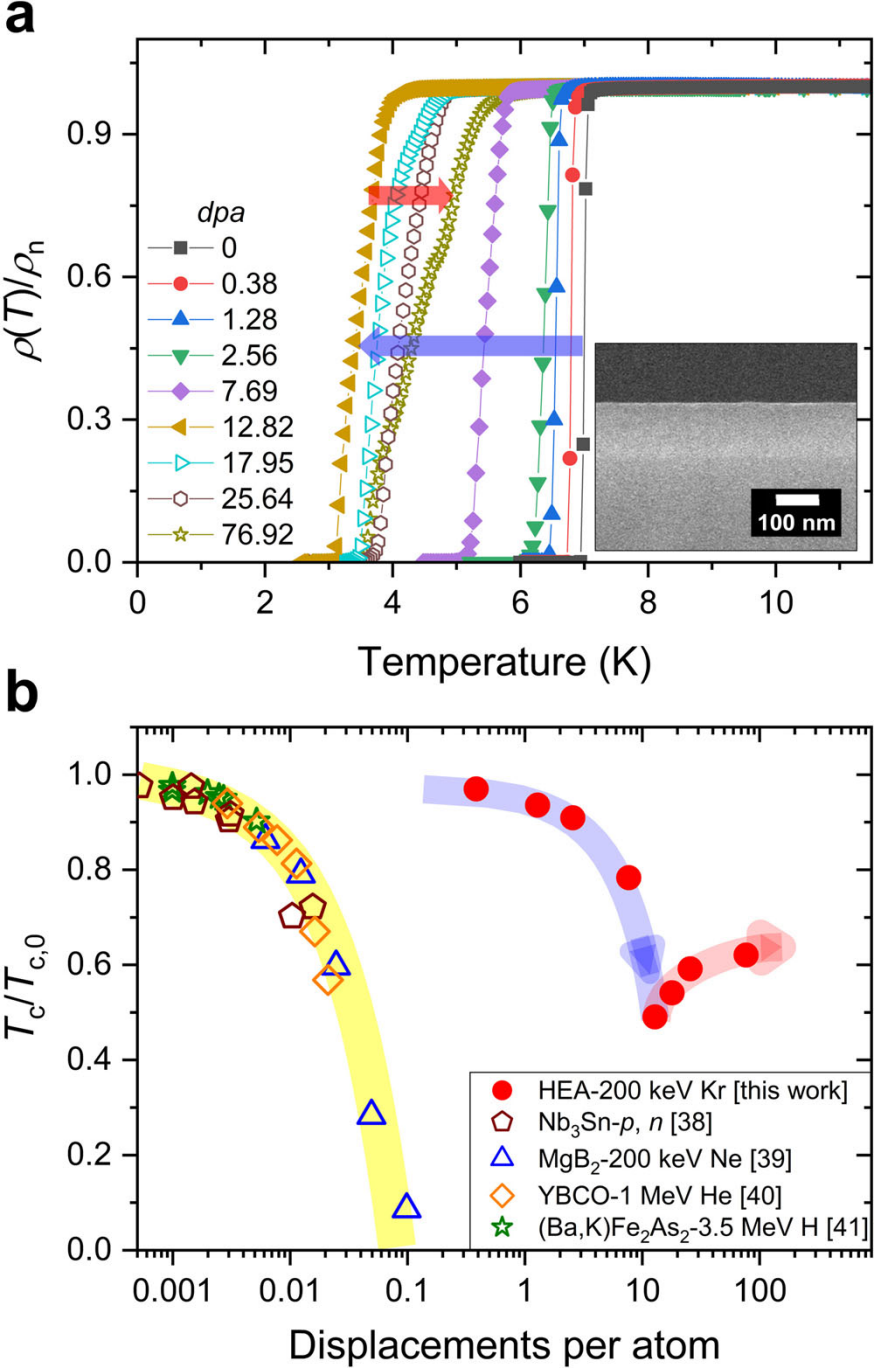
Changes in SC transition temperature for HEA SC thin films against irradiation damage. **a** *ρ*(*T*) curves near *T*_c_ for the 200 keV Kr-ion-irradiated Ta–Nb–Hf–Zr–Ti HEA SC thin films with a thickness of 115 nm, where *ρ*(*T*) was normalized by *ρ*_n_ for comparison. The *T*_c_ of HEA SC thin films decreases gradually with an increase in *dpa*, but for *dpa* > 12.82 (left triangles), strikingly, the *T*_c_ exhibits an increase despite the increase in the *dpa* level (see the arrow direction). **b** *T*_c_/*T*_c,0_ with respect to *dpa* for representative SC materials with potential for practical applications, where *T*_c,0_ represents the *T*_c_ of the pristine sample and *p* and *n* for Nb_3_Sn indicate proton irradiation and neutron irradiation, respectively. The Ta–Nb–Hf–Zr–Ti HEA SC thin films show a remarkably robust superconductivity against irradiation-induced disorder compared with that of the other SC materials. Half-transparent shades are guides to the eyes.

Robust superconductivity with respect to disorder has been proposed on the basis of experimental observations of the insensitivity of the *T*_c_ of HEA superconductors to the disorder introduced by different levels of constituent atoms^16,34^. In addition, the stable superconductivity of HEAs containing the radioactive element U makes HEA superconductors promising for application in heavy-irradiation environments, such as in aerospace applications and nuclear fusion^6,7^. However, to the best of our knowledge, no studies have investigated the effect of ion irradiation on HEA superconductors. To examine the stability of the SC phase of HEAs against irradiation damage, high-quality HEA SC thin films were systematically irradiated with low-energy 200 keV Kr ions^35–37^. Figure 4a shows the *ρ*(*T*) curves for the irradiated Ta–Nb–Hf–Zr–Ti HEA SC thin films with a thickness of 115 nm, where *ρ*(*T*) was normalized by *ρ*_n_ for comparison. The inset of Fig. 4a presents a cross-sectional SEM image used for ion irradiation. The disorder levels were adjusted using amounts of irradiated Kr ions from 1.5 × 10^14^ to 3 × 10^16^ Kr ions cm^−2^, corresponding to displacements per atom (*dpa*) from 0.38 to 76.92 (see Supplementary Fig. 8). The superconductivity of the HEA SC thin film was remarkably resistant to the displacement damage produced by irradiation. The *T*_cR_ of 7.0 K for the pristine film decreased gradually as the level of *dpa* increased, but remained at 3.4 K at *dpa* = 12.82. For further increases in the *dpa* level, strikingly, the *T*_cR_ showed an increase rather than a decrease and reached 4.3 K at the extreme damage level of *dpa* = 76.92.

Figure 4b shows a plot of *T*_c_ as a function of *dpa* for Ta–Nb–Hf–Zr–Ti HEA SC thin films and other superconductors with potential for practical application, such as Nb_3_Sn^38^, MgB_2_^39^, YBa_2_Cu_3_O_7−*x*_^40^, and K-doped BaFe_2_As_2_^41^. Here, *T*_c_ was normalized to the *T*_c_ of each pristine sample (*T*_c,0_) for comparison. With an increase in the *dpa* value, *T*_c_ decreased monotonically and is expected to be completely suppressed below *dpa* = 1 for the other superconductors. However, the range of the *dpa* values for the HEA SC thin films differed significantly from those for the other superconductors: the superconductivity of the HEA SC thin films was over 1000 times more resistant to displacement damage than that of the other superconductors. The suppression of *T*_c_ in crystalline SC materials against *dpa* is generally considered to be a result of the disorder effect associated with SC gap pairing symmetry and the degradation of crystallinity due to atomic disorder^38,42^. In contrast, the extraordinarily stable SC phase of HEAs against irradiation-induced disorder could be related to the occurrence of amorphization-recrystallization and the high interstitial–vacancy recombination rate associated with the high atomic-level stress and/or high-level lattice distortion in HEAs resulting from their high configuration entropy^3,43–45^. In addition, the random occupation of lattice sites of the constituent atoms of HEAs seems related to the robust superconductivity of HEAs against irradiation damage, because the atomic positions in the lattice altered by the recombination may have little effect on the characteristic of HEAs^16^. Our irradiation study of Ta–Nb–Hf–Zr–Ti HEA SC thin films manifests the excellent robustness of the SC phase against irradiation damage, which is expected to open the door to many potential applications of HEA superconductors.

In summary, we fabricated high-quality Ta–Nb–Hf–Zr–Ti HEA SC thin films using the PLD method. A large critical current density of *J*_c_ > 1 MA cm^−2^ was realized, and a *J*_c_ of 0.1 MA cm^−2^—a common benchmark for large-scale application—was maintained under magnetic fields of approximately 3.4 and 2.0 T at 2.0 and 4.2 K, respectively. In addition, the superconductivity of the HEA SC thin films was over 1000 times more robust to displacement damage than that of other representative SC materials, such as Nb_3_Sn, MgB_2_, Fe-based superconductors, and high-*T*_c_ cuprates. Taken together with the wide range of growth temperatures for high-quality thin films, these results demonstrate the great potential of HEA superconductors for practical applications under various extreme environments, such as aerospace applications, high-field SC magnets, and irradiation environments.

## Methods

### Fabrication of Ta–Nb–Hf–Zr–Ti HEA SC thin films

A Ta_1/6_Nb_2/6_Hf_1/6_Zr_1/6_Ti_1/6_ HEA SC target with a diameter of 15 mm and bulk samples A and B were prepared using a planetary ball-milling (FRITSCH GmbH, PULVERISETTE 5, Germany) and hot-press sintering process^3^. Ta (99.98%), Nb (99.8%), Hf (99.6%), Zr (99.5%), and Ti (99.5%) powders with a compositional ratio of 1:2:1:1:1 were loaded in a stainless-steel jar equipped with a stainless-steel ball in an Ar atmosphere to prevent oxidation. Ball milling with a ball to HEA powder ratio of 10: 1 was performed at a rotation speed of 400 rpm for 24 h using a planetary ball milling machine, after which the powder was sintered via a hot-press sintering method at 1000 °C for 1 h under a uniaxial pressure of 50 MPa.

High-quality Ta–Nb–Hf–Zr–Ti HEA SC thin films were fabricated on *c*-cut Al_2_O_3_ substrates (10 mm × 10 mm) using a PLD technique. The laser beam was generated using a KrF excimer laser (λ = 248 nm, IPEX864; LightMachinery) and the thin films were deposited using a laser energy density of ~3.85 J cm^−2^ and a repetition rate of 10 Hz in a high vacuum state of ~10^−6^ Torr. Thin films were fabricated with substrate temperatures (*T*_s_) ranging from 270 to 620 °C, and all the films exhibited superconductivity. By controlling the growth time, we obtained Ta–Nb–Hf–Zr–Ti HEA SC thin films with thicknesses in the range of 115–700 nm.

### Ion irradiation and characterization of thin films

Kr ions with a beam energy of 200 keV were used to irradiate Ta–Nb–Hf–Zr–Ti HEA SC thin films with a thickness of 115 nm at the Korea Multi-Purpose Accelerator Complex (KOMAC) in Gyeongju. Kr ion levels of 1.5 × 10^14^, 5 × 10^14^, 1 × 10^15^, 3 × 10^15^, 5 × 10^15^, 7 × 10^15^, 1 × 10^16^, and 3 × 10^16^ Kr ions cm^−2^ were irradiated onto the films at room temperature, with a tilt angle of 7° to avoid channeling effects during the irradiation. The displacement damage produced in the Ta_1/6_Nb_2/6_Hf_1/6_Zr_1/6_Ti_1/6_ HEA superconductor (density of 9.9 g cm^−3^) by the 200 keV Kr-ion irradiation was estimated using the Stopping and Range of Ions in Matter (SRIM) Monte Carlo simulation program with averaged displacement threshold energy values of 90 eV (Ta), 78 eV (Nb), 61 eV (Hf), 40 eV (Zr), and 30 eV (Ti)^46,47^. The simulated target displacement values from the SRIM program were converted into displacements per atom (*dpa*) values using the following relationship (see also Supplementary Fig. 8):

2 $$\frac{{{{{{\rm{displacements}}}}}}}{{{{{{\rm{atom}}}}}}\times {{{{\AA}} }}}\times \frac{{10}^{8}({{{{\AA}} }}/{{{{{\rm{cm}}}}}})\times {{{{{\rm{dose}}}}}}\left({{{{{\rm{atoms}}}}}}/{{{{{{\rm{cm}}}}}}}^{2}\right)}{{\rho }_{{{{{{\rm{HEA}}}}}}}\left(\frac{{{{{{\rm{atoms}}}}}}}{{{{{{{\rm{cm}}}}}}}^{3}}\right)}=\frac{{{{{{\rm{displacements}}}}}}}{{{{{{\rm{atom}}}}}}}={dpa},$$

where *ρ*_HEA_ = 5.226 × 10^22^ atoms cm^−3^ is the atomic density of Ta_1/6_Nb_2/6_Hf_1/6_Zr_1/6_Ti_1/6_. The doses 1.5 × 10^14^, 5 × 10^14^, 1 × 10^15^, 3 × 10^15^, 5 × 10^15^, 7 × 10^15^, 1 × 10^16^, and 3 × 10^16^ Kr ions cm^−2^ corresponded to *dpa* values of 0.38, 1.28, 2.56, 7.69, 12.82, 17.95, 25.64, and 76.92, respectively.

The crystal structure of the fabricated Ta–Nb–Hf–Zr–Ti HEA SC thin films was investigated using an X-ray diffractometer (Rigaku miniflex-600 diffractometer, Cu-K_α1_ radiation, λ = 1.541 Å). The thicknesses and compositional ratios of the thin films were examined using scanning electron microscope and energy-dispersive X-ray spectrometry, respectively. The SC transition temperature (*T*_c_) of the fabricated Ta–Nb–Hf–Zr–Ti HEA SC thin films was evaluated from the temperature dependence of the electrical resistivity (*ρ*) and magnetization (*M*) using a physical property measurement system (PPMS 9 T, Quantum Design) and a magnetic property measurement system (MPMS 5 T, Quantum Design), respectively. The *ρ*(*T*) was measured using the standard four-probe method with an Au coating on the four-point contact regions to achieve good ohmic contact, and the upper critical field (*μ*_0_*H*_c2_) was estimated by measuring the *ρ*(*T*) curve in the magnetic field from 0 to 9 T in the PPMS. The critical current density (*J*_c_) of the HEA SC thin films as a function of the magnetic field was calculated from the magnetization hysteresis (*M–H*) loops based on Bean’s critical state model (*J*_c_ = 15Δ*M/rV*) (see Supplementary Fig. 7). Here, the *M–H* loops were measured using MPMS, and Δ*M* is the difference in *M* values at the same magnetic field in the *M*–*H* loops, *V* is the volume of the film, and *r* is the radius corresponding to the total area of the surface of the film^48^. The direction of the magnetic field was perpendicular to the *ab* plane of the film for *H*_c2_(*T*) and *J*_c_(*H*).

## Supplementary information

Supplementary Information

## Data Availability

The authors declare that all the data supporting the finding of this study are available within this article and its Supplementary Information files and are available from the corresponding author on reasonable request.
