# Supplementary material for: High critical current density and high-tolerance superconductivity in high-entropy alloy thin films
Source: Nat Commun. 2022 Jun 11;13:3373. doi: 10.1038/s41467-022-30912-5 (PMC9188561; doi:10.1038/s41467-022-30912-5)
Supplement: Supplementary file 1 — Supplementary Information [file 41467_2022_30912_MOESM1_ESM.pdf]

Supplementary Information for "**High critical current density and high-tolerance superconductivity in high-entropy alloy thin films**"

Soon-Gil Jung[1,2,6,*], Yoonseok Han[1,2,6], Jin Hee Kim[3,4,6], Rahmatul Hidayati[3,4], Jong-Soo Rhyee[3,4], Jung Min Lee[2], Won Nam Kang[2], Woo Seok Choi[2], Hye-Ran Jeon[5], Jaekwon Suk[5], and Tuson Park[1,2,*]

[1] Center for Quantum Materials and Superconductivity (CQMS), Sungkyunkwan University, Suwon 16419, Republic of Korea

[2] Department of Physics, Sungkyunkwan University, Suwon 16419, Republic of Korea

[3] Department of Applied Physics, Integrated Education Institute for Frontier Science and Technology (BK 21 Four), Kyung Hee University, Yongin 17104, Republic of Korea

[4] Institute of Natural Science, Kyung Hee University, Yongin 17104, Republic of Korea

[5] Korea Multi-purpose Accelerator Complex, Korea Atomic Energy Research Institute, Gyeongju, Gyeongbuk 38180, Republic of Korea

*Corresponding author. Email: prosgjung@gmail.com (S.–G.J.); tp8701@skku.edu (T.P.)

In this supplement, we present additional data and analyses that support the results presented in the main text.

1. Supplementary Table 1

Supplementary Table 1. Atomic ratio of Ta–Nb–Hf–Zr–Ti HEA superconducting (SC) thin films.

2. Supplementary Figures 1 to 8

Supplementary Figure 1. EDS results for Ta–Nb–Hf–Zr–Ti HEA target.

Supplementary Figure 2. EDS results for Ta–Nb–Hf–Zr–Ti HEA SC thin films.

Supplementary Figure 3. SC transition temperature (T_c) with respect to the valence electron count (VEC) for Ta–Nb–Hf–Zr-Ti HEA superconductors.

Supplementary Figure 4. Temperature dependence of electrical resistance in a magnetic fields for Ta–Nb–Hf–Zr–Ti HEA SC thin films.

Supplementary Figure 5. Temperature dependence of magnetization and magnetic field dependence of critical current density for HEA superconducting bulk samples and thin films.

Supplementary Figure 6. Flux pinning force density of Ta–Nb–Hf–Zr–Ti HEA SC thin films.

Supplementary Figure 7. Magnetization hysteresis loops for Ta–Nb–Hf–Zr–Ti HEA SC thin films.

Supplementary Figure 8. Displacements simulated by the Stopping and Range of Ions in Matter (SRIM) software for 200-keV Kr-ion-irradiated $Ta_{1/6}Nb_{2/6}Hf_{1/6}Zr_{1/6}Ti_{1/6}$.

3. Supplementary References

1. Supplementary Table 1

Supplementary Table 1. **Atomic ratio of Ta–Nb–Hf–Zr–Ti HEA superconducting (SC) thin films.** Results obtained from energy dispersive spectroscopy (EDS) for the atomic ratios of Ta–Nb–Hf–Zr–Ti HEA target and SC thin films fabricated via the pulsed laser deposition (PLD) technique at various substrate temperatures (T_s): 270, 370, 470, 500, 520, 540, 570, and 620 ºC. No significant differences were observed among the compositional ratios of the films. The deviation of the chemical composition ratio between the target and the HEA thin film is related to the relatively larger sputtering yield of Ti and Zr atoms than other constituent elements [S1,S2]. The similar atomic ratios of each HEA film, regardless of T_s, indicates that the sticking coefficient of each element is not significantly different.

T_s / Element	Target	270 ºC	370 ºC	470 ºC	500 ºC	520 ºC	540 ºC	570 ºC	620 ºC
Ti	14.78	11.04	12.59	12.86	11.86	11.70	12.26	11.92	12.58
Zr	18.57	14.24	14.26	12.98	12.42	11.84	11.53	13.99	15.38
Hf	16.86	18.11	17.66	17.77	18.55	18.23	18.25	18.09	16.57
Nb	33.37	37.31	36.95	36.80	37.36	38.32	38.44	36.95	36.81
Ta	16.42	19.30	18.54	19.59	19.82	19.91	19.52	19.05	18.66

2. Supplementary Figures 1 to 8

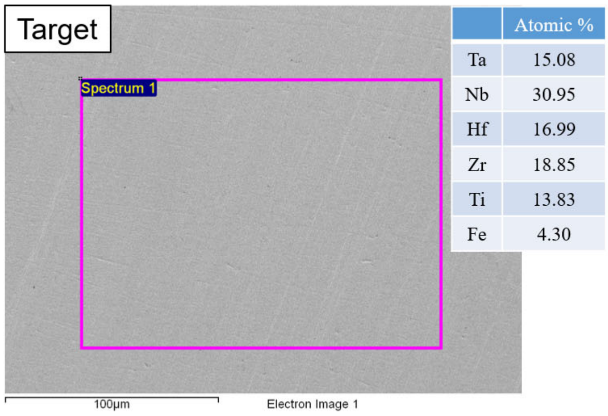

	Atomic %
Ta	15.08
Nb	30.95
Hf	16.99
Zr	18.85
Ti	13.83
Fe	4.30

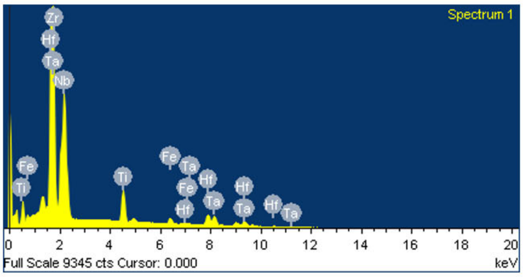

Supplementary Figure 1. EDS results for Ta–Nb–Hf–Zr–Ti HEA target. EDS spectra of HEA target used for the deposition of HEA SC thin films in this study. Except the constituent atoms of Ta–Nb–Hf–Zr–Ti HEA superconductors, the additional peaks in the EDS analysis are related to Fe impurity generated during the mechanical processing of ball milling. In general, high-energy ball milling processes often produce additional impurities from the milling tools [S3]. Stainless steel was used for the milling tool in this study, but Cr and Ni impurities were not detected within the EDS resolution.

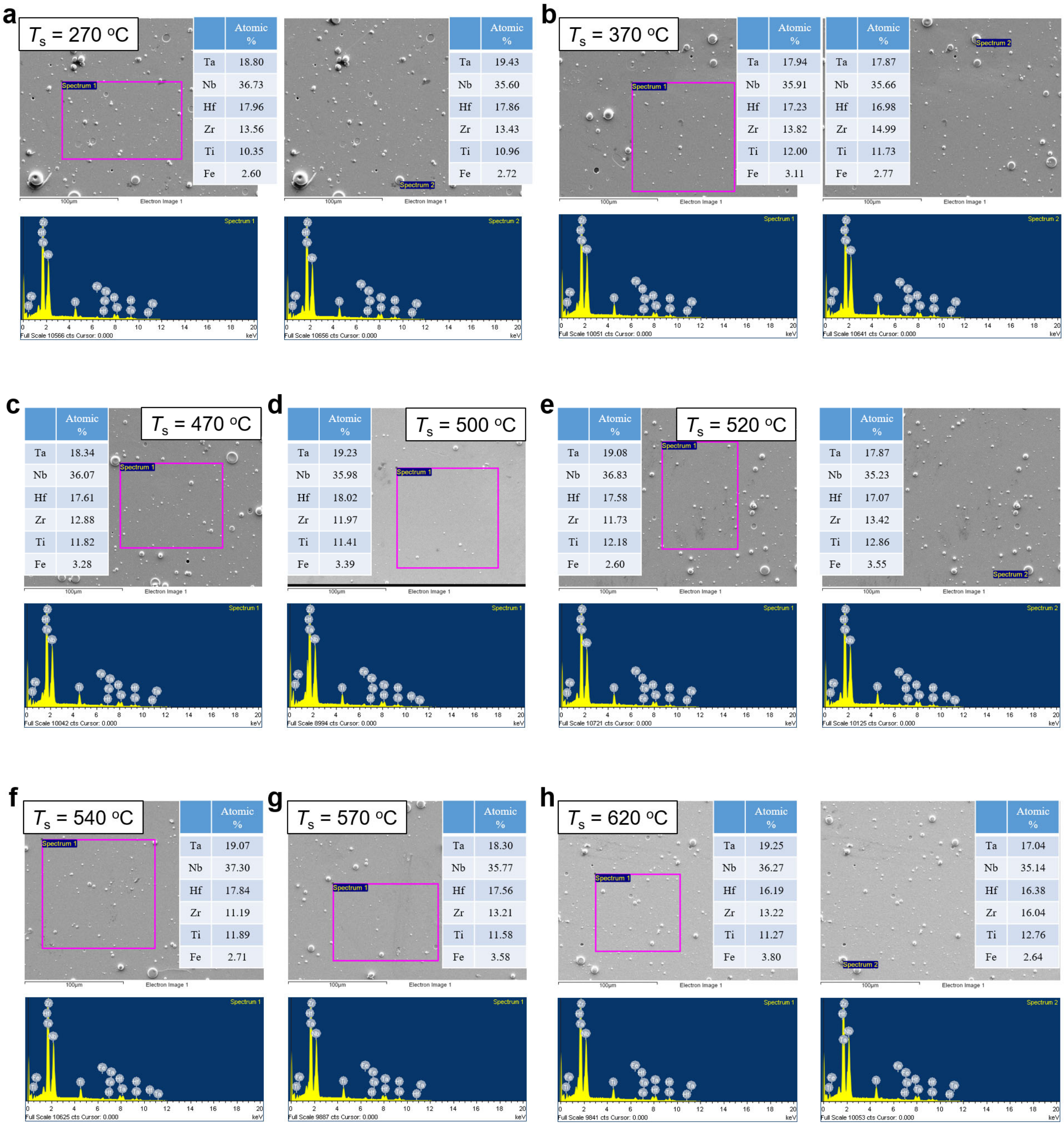

Supplementary Figure 2. EDS results for Ta–Nb–Hf–Zr–Ti HEA SC thin films. a–h, EDS spectra of Ta–Nb–Hf–Zr–Ti HEA SC thin films fabricated at T_s = 270–620 ºC for a large area and a droplet region. The difference in atomic ratios between the large area and the droplet region is negligible. Here, the peaks of elements C, O, and Al due to general contamination and Al_2O_3 substrate were not indicated.

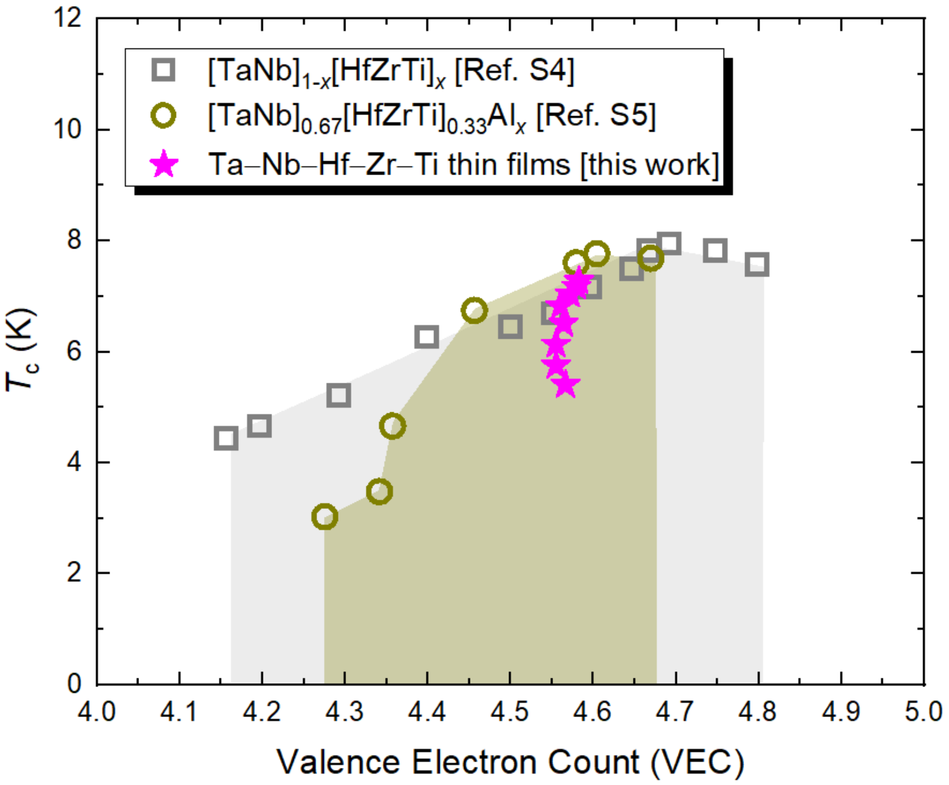

Supplementary Figure 3. SC transition temperature (T_c) with respect to the valence electron count (VEC) for Ta–Nb–Hf–Zr-Ti HEA superconductors. The range of VEC values, i.e., electrons per atom (*e/a*, *d*-electron), for the Ta–Nb–Hf–Zr–Ti HEA SC thin films was 4.55–4.58, which is close to the optimal VEC for the highest T_c in HEA superconductors with a bcc structure [S4–S6], although their T_c values are different. This result indicates that the substrate temperature T_s is one of the critical parameters for fabricating high-quality HEA SC thin films. The VEC value represents the average of the number of valence electrons for the constituent atoms that compose the alloy. For example, the number of valence electrons is 5 and 4 for (Ta, Nb) and (Hf, Zr, Ti), respectively. Here, the VEC value of the Ta–Nb–Hf–Zr–Ti HEA SC thin films was determined using the EDS results in Supplementary Table 1.

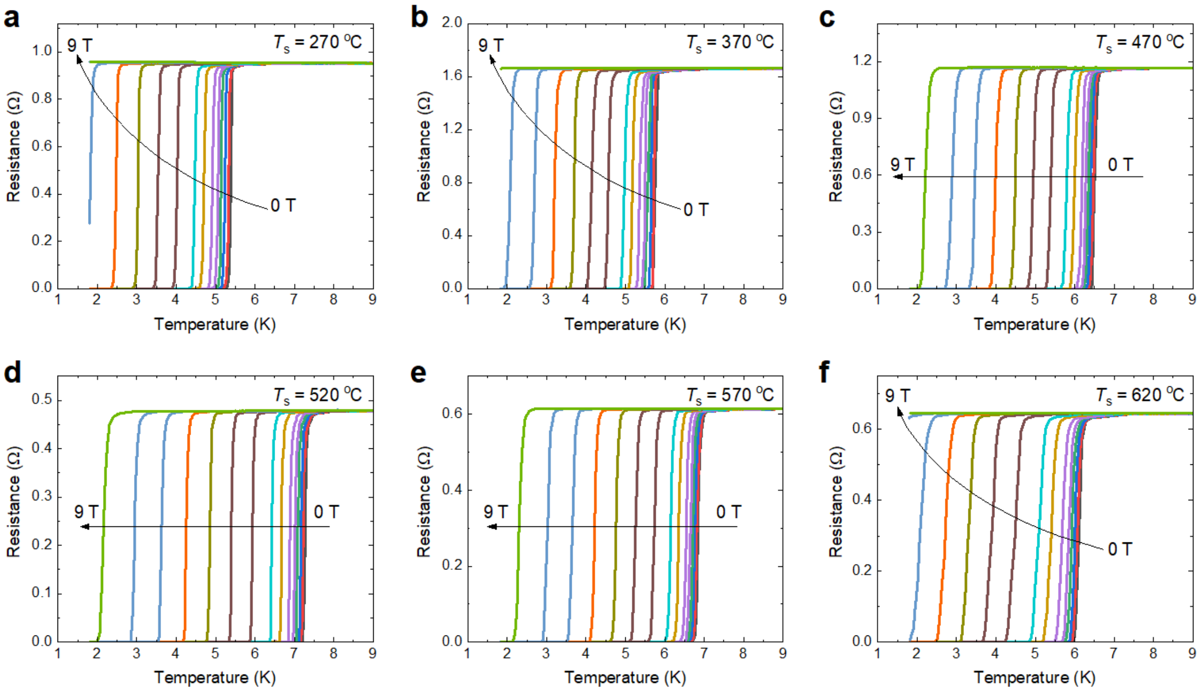

Supplementary Figure 4. Temperature dependence of electrical resistance in a magnetic fields for Ta–Nb–Hf–Zr–Ti HEA SC thin films. a–f, Electrical resistance as a function of temperature at magnetic fields from 0 to 9 T for the HEA SC thin films fabricated at T_s = 270, 370, 470, 520, 570, and 620 ºC. $H_{c2}(T)$ was obtained using the criterion of T_{cR}, namely 50% transition of the resistance at the T_c onset for each magnetic field.

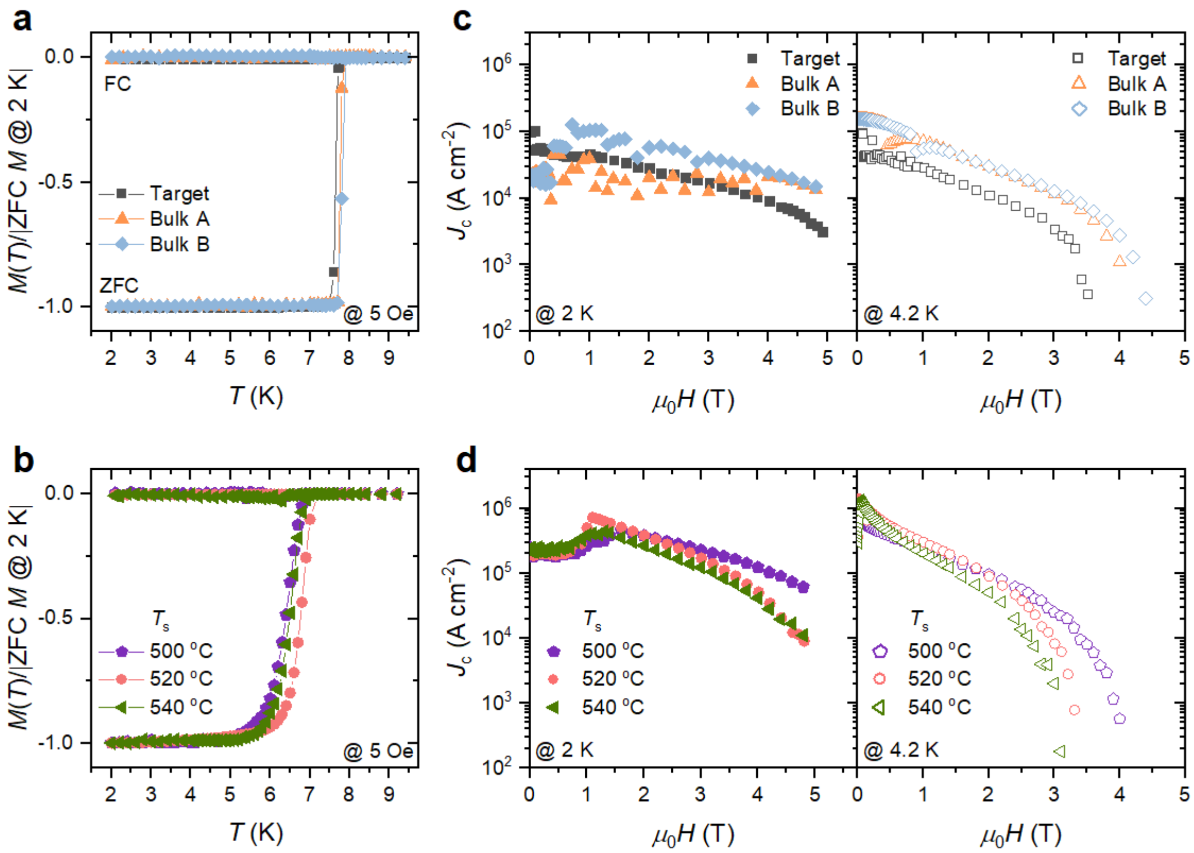

Supplementary Figure 5. Temperature dependence of magnetization and magnetic field dependence of critical current density for HEA superconducting bulk samples and thin films. Zero-field-cooled (ZFC) and field-cooled (FC) magnetization (*M*) for (**a**) HEA bulk samples and (**b**) SC thin films. Here, *M*(*T*) was normalized by the absolute ZFC *M* value at 2 K for comparison. $Ta_{1/6}Nb_{2/6}Hf_{1/6}Zr_{1/6}Ti_{1/6}$ HEA superconductors used as the target for growing HEA SC thin films show a very sharp SC transition in the ZFC *M*(*T*) curve with T_{cM} of 7.8 K. **c,** The $J_c(H)$ for HEA bulk samples with similar composition ratio to the thin films, where the atomic percent of Ta, Nb, Hf, Zr, Ti for the bulk A and B are 19.96, 39.47, 19.46, 12.32, 10.79 and 19.80, 38.54, 17.23, 12.71, 11.72, respectively. Interestingly, T_c and J_c of bulk A and B are higher than the $Ta_{1/6}Nb_{2/6}Hf_{1/6}Zr_{1/6}Ti_{1/6}$ target. Strong fluctuations in the $J_c(H)$ of bulk A and B are caused by a large flux jump [S7,S8]. (**d**) HEA SC thin films deposited at T_s = 500, 520, and 540 ºC. All the films show considerably larger J_c values than the bulk samples. In addition, the film with the highest T_c, deposited at 520 ºC, shows the highest J_c at low fields, whereas the J_c of the film fabricated at 500 ºC has better field performance at high fields.

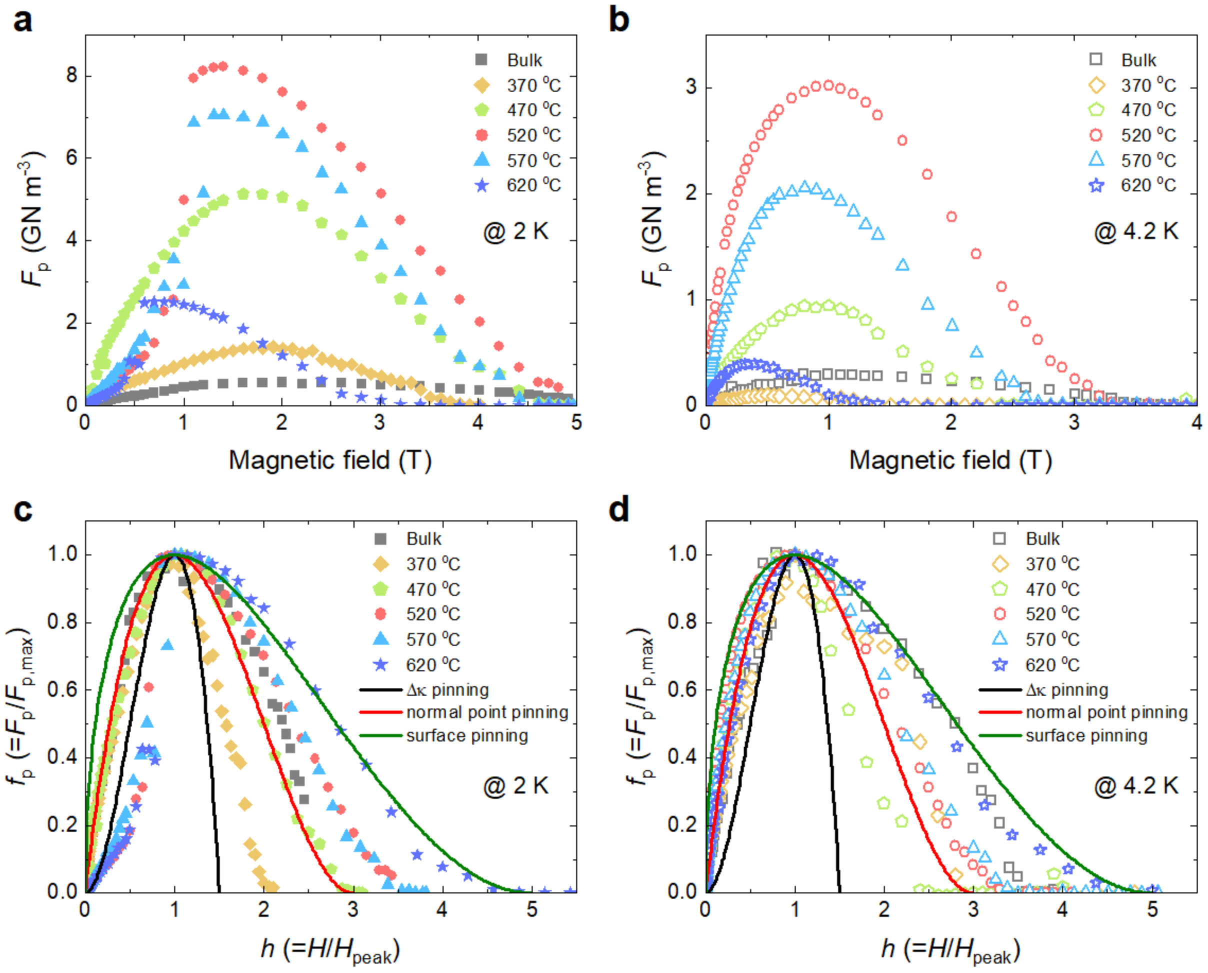

Supplementary Figure 6. Flux pinning force density of Ta–Nb–Hf–Zr–Ti HEA SC thin films. Magnetic field dependence of the volume flux pinning force density ($F_p = J_c \times \mu_0 H$) for the HEA SC bulk and thin films at (**a**) 2.0 K and (**b**) 4.2 K. The maximum F_p ($F_{p,max}$) values of the film deposited at the optimal T_s = 520 °C are 8.22 and 3.02 GN m^{-3} at 2.0 and 4.2 K, which are 14.7 and 10.4 times higher than that of the bulk sample, respectively. **c**, **d**, Normalized flux pinning force density ($F_p/F_{p,max}$) as a function of the reduced magnetic field (H/H_{peak}) at 2.0 and 4.2 K, respectively, where H_{peak} represents the magnetic field at $F_{p,max}$. The flux pinning mechanism of most of the films is close to normal point pinning, indicating that the intrinsic disorder associated with the different atomic sizes of the constituent atoms in HEAs plays an important role in the field performance of J_c in HEA superconductors. The solid lines were plotted using the equations $f_p(h) = 3h^2(1\text{-}2h/3)$, $(9/4)h(1\text{-}h/3)^2$, and $(25/16)h^{1/2}(1\text{-}h/5)^2$ for $\Delta\kappa$-pinning, normal point pinning, and surface pinning, respectively, where κ is the Ginzburg–Landau parameter [S9–S11].

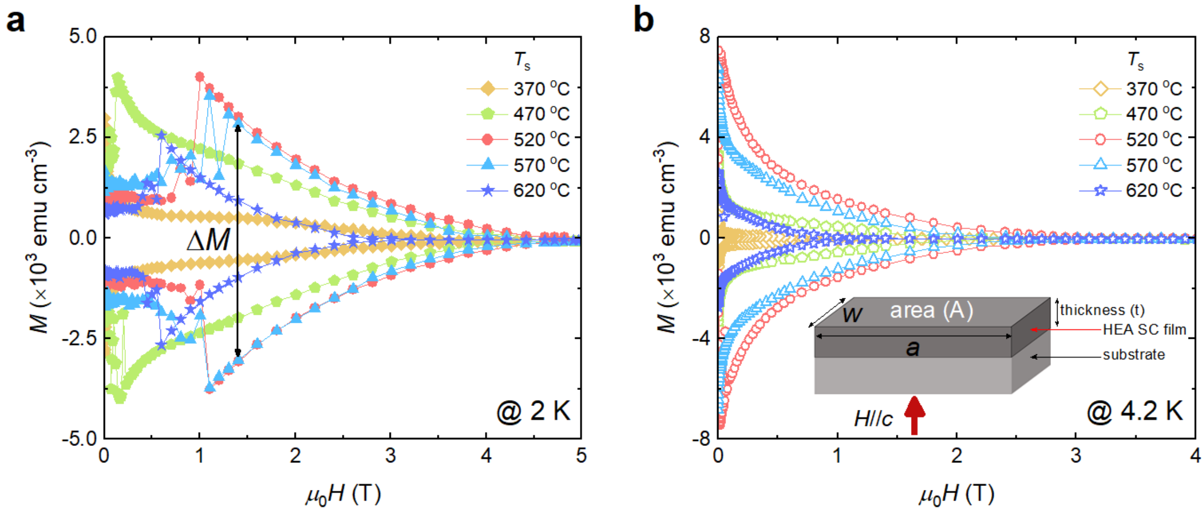

Supplementary Figure 7. Magnetization hysteresis loops for Ta–Nb–Hf–Zr–Ti HEA SC thin films. Magnetic field–magnetization (M–H) hysteresis loops of Ta–Nb–Hf–Zr–Ti HEA SC thin films at (**a**) 2.0 K and (**b**) 4.2 K. A large flux jump was observed at 2.0 K for the HEA SC thin films with a relatively higher T_c, which resulted in a smaller low-field J_c compared with that at 4.2 K. The flux jump is attributed to thermomagnetic instability [S7,S8]; thus, the J_c value of an HEA SC thin film can be increased by reducing the thermal instability. The magnetic field dependence of J_c can be estimated from the M–H loops by using Bean's critical state model ($J_c = 30\Delta M/d$). Here, ΔM is the difference of M value at the same magnetic field in the irreversible regions, as indicated in Supplementary Fig. 7a, and d is the corresponding diameter of the total area, $A = \pi(d/2)^2$, of the film's surface, as described in the inset of Supplementary Fig. 7b. The $J_c(H)$ for all HEA SC thin films presented in this study was determined from the above relation. On the other hand, the J_c for the bulk sample with a rectangular shape is generally estimated from the relation $J_c = 20\Delta M/[w(1\text{-}w/3a)]$, where w and a is width and length, respectively, for $w < a$ [S12,S13].

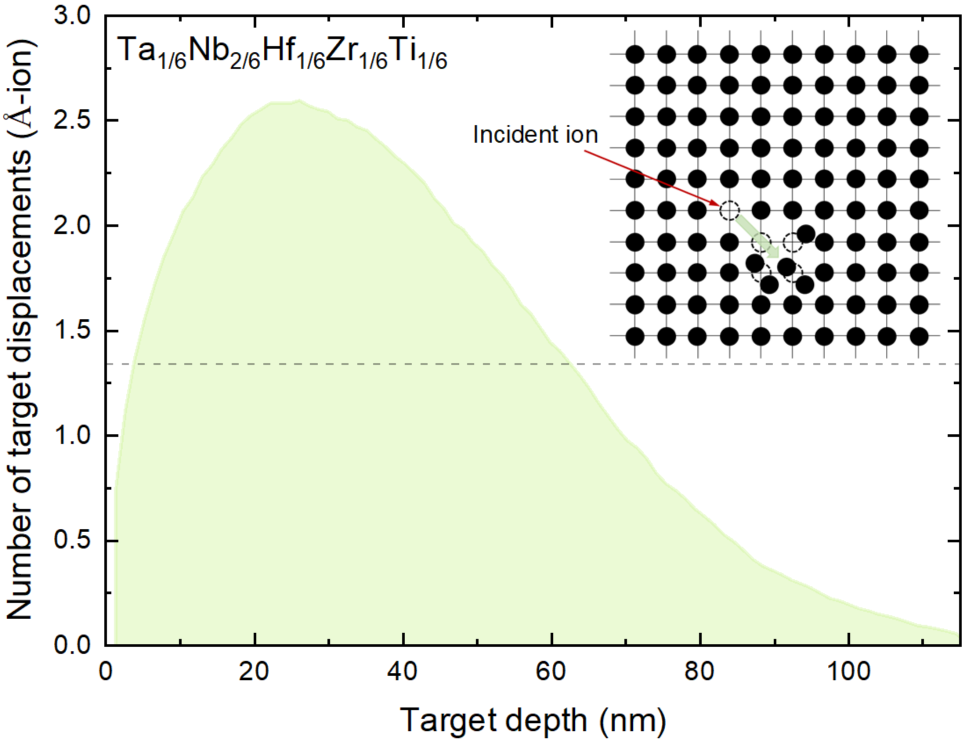

Supplementary Figure 8. Displacements simulated by the Stopping and Range of Ions in Matter (SRIM) software for 200-keV Kr-ion-irradiated $Ta_{1/6}Nb_{2/6}Hf_{1/6}Zr_{1/6}Ti_{1/6}$. SRIM simulation result for the number of displacements produced by 200-keV Kr-ion irradiation for a $Ta_{1/6}Nb_{2/6}Hf_{1/6}Zr_{1/6}Ti_{1/6}$ HEA (with a mass density of 9.9 g cm^{-3} and an atomic density of ρ_{HEA} = 5.226 × 10^{22} atoms cm^{-3}). The *dpa* value was obtained as follows:

$$\frac{\text{displacements}}{\text{atom}\times\text{Å}} \times \frac{10^8(\text{Å/cm})\times\text{dose}(\text{atoms/cm}^2)}{\rho_{\text{HEA}}(\frac{\text{atoms}}{\text{cm}^3})} = \frac{\text{displacements}}{\text{atom}} = dpa. \quad (1)$$

Here, the *dpa* values were evaluated using the average number of displacements (1.34 Å-ion), which is indicated by the dashed line. The doses of 1.5 × 10^{14}, 5 × 10^{14}, 1 × 10^{15}, 3 × 10^{15}, 5 × 10^{15}, 7 × 10^{15}, 1 × 10^{16}, and 3 × 10^{16} Kr ions cm^{-2} corresponded to *dpa* values of 0.38, 1.28, 2.56, 7.69, 12.82, 17.95, 25.64, and 76.92, respectively. The *dpa* value reflects the level of displacement damage generated by irradiation, and *dpa* = 0.01 indicates that 1% of target atoms were displaced from their lattice sites by irradiation. Inset is a simple illustration showing an example corresponding to *dpa* = 0.05 on a 10 × 10 two-dimensional square lattice.

3. Supplementary References

[S1] Schou, J., Physical aspects of the pulsed laser deposition technique: The stoichiometric trans fer of material from target to film. *Appl. Surf. Sci.* **255**, 5191-5198 (2009).

[S2] Behrisch, R. & Eckstein, W. (Eds.), Sputtering by Particle Bombardment: Experiments and Computer Calculations from Threshold to MeV Energies Springer, 2007).

[S3] Suryanarayana, C., Mechanical alloying and milling. *Prog. Mater. Sci.* **46**, 1-184 (2001).

[S4] von Rohr, F. O., Winiarski, M. J., Tao, J., Klimczuk, T. & Cava, R. J., Effect of electron count and chemical complexity in the Ta–Nb–Hf–Zr–Ti high-entropy alloy superconductor. *PNAS* **113**, E7144–E7150 (2016).

[S5] von Rohr, F. O. & Cava, R. J., Isoelectronic substitutions and aluminium alloying in the Ta–Nb–Hf–Zr–Ti high-entropy alloy superconductor. *Phys. Rev. Mater.* **2**, 034801 (2018).

[S6] Sun, L. & Cava, R. J., High-entropy alloy superconductors: Status, opportunities, and challenges. *Phys. Rev. Mater.* **3**, 090301 (2019).

[S7] Choi, E. –M., Lee, H. –S., Kim, H. –J., Lee, S. –I., Kim, H. –J. & Kang, W. N., Enhancement at low temperatures of the critical current density for Au-coated MgB_2 thin films. *Appl. Phys. Lett.* **84**, 82–84 (2004).

[S8] Mints, R. G. & Brandt, E. H., Flux jumping in thin films. *Phys. Rev. B* **54**, 12421–12426 (1996).

[S9] Dew-Hughes, D., Flux pinning mechanisms in type II superconductors. *Philos. Mag.* **30**, 293–305 (1974).

[S10] Higuchi, T., Yoo, S. I. & Murakami, M., Comparative study of critical current densities and flux pinning among a flux-grown $NdBa_2Cu_3O_y$ single crystal, melt-textured Nd–Ba–Cu–O, and Y–Ba–Cu–O bulks. *Phys. Rev. B* **59**, 1514–1527 (1999).

[S11] Jung, S. –G., Pham, D., Han, Y., Lee, J. M., Kang, W. N., Kim, C., Yeo, S., Jun, B. –H. & Park, T., Improvement of bulk superconducting current capability of MgB_2 films using surface degradation. *Scr. Mater.* **209**, 114424 (2022).

[S12] Kim, H. –J., Kang, W. N., Choi, E. –M., Kim, M. –S, Kim, K. H. P. & Lee, S. –I., High current-carrying capability in *c*-axis-oriented superconducting MgB_2 thin films. *Phys. Rev. Lett.* **87**, 087002 (2001).

[S13] Gyorgy, E. M., van Dover, R. B., Jackson, K. A., Schneemeyer, L. F. & Waszczak, J. V., Anisotropic critical currents in $Ba_2YCu_3O_7$ analyzed using an extended Bean model. *Appl. Phys. Lett.* **55**, 283-285 (1989).
